# The impact of environmental regulations on carbon emissions in countries with different levels of emissions

**DOI:** 10.1007/s11356-024-35702-8

**Published:** 2024-12-06

**Authors:** Justyna Borowiec, Monika Papież, Sławomir Śmiech

**Affiliations:** Department of Statistics, Krakow University of Economics, Rakowicka 27, 31-510 Cracow, Poland

**Keywords:** Environmental regulations; market, Based and non, Market, Based instruments, Technology support policies, MMQR – moments quantile panel regression model, ND-GAIN Country Index

## Abstract

The study analyzes the impact of environmental regulations on carbon emissions in countries with different levels of emissions, utilizing two measures of carbon emissions based on: production (PBA) and consumption (CBA) accounting. Environmental regulations are measured by means of three components of the Environmental Policy Stringency (EPS) index: market-based and non-market-based instruments, and technology support. The Moments-Quantile Regression method is employed to assess the effectiveness of these policies across countries with varying levels of emissions—high, medium, and low within the Environmental Kuznets Curve. The findings indicate that increased stringency in environmental regulations correlates with reduced carbon emissions per capita. Notably, the EPS index has a more significant effect on reducing PBA emissions compared to CBA emissions. A key finding is that the EPS index is more effective in countries with lower pollution per capita (i.e., lower quantiles) than in those with higher pollution per capita. Among the three components, market-based instruments are identified as the most effective in reducing carbon emissions. Additionally, in countries where per capita emissions are relatively low, the combination of market and non-market instruments proves to be the most effective in reducing emissions. In contrast, the highest carbon emitters per capita tend to achieve emissions reductions primarily through technological support.

## Introduction

Global warming and climate change are among the most critical issues confronting the world today. At the heart of the problem are greenhouse gas (GHG) emissions, with CO_2_ being the most significant contributor. These emissions drive the greenhouse effect, which accelerates climate change. Addressing this challenge requires coordinated global action to transition toward sustainable, low-carbon economies (Dagoumas and Barker 2010). The political discourse on climate change, which began over three decades ago, led to key milestones such as the Kyoto Protocol (1997), an extension of the 1992 United Nations Framework Convention on Climate Change (UNFCCC), and the Paris Agreement (2015), which urges countries to commit to reducing GHG emissions. Despite these efforts, some nations, particularly developing ones, have been slower to prioritize environmental objectives.

As highlighted by Hashmi and Alam (2019) and Wolde-Rufael and Mulat-Weldemeskel (2021), public authorities rely on two main strategies to combat climate change: promoting green innovation and implementing environmental policies. These policies include carbon regulations and environmental taxes, such as carbon pricing.

The literature presents two contrasting views on the effect of stringent environmental regulations on pollution. The Pollution Haven Hypothesis (PHH), first proposed by Copeland and Taylor (1994), suggests that strict environmental regulations in developed countries lead to the relocation of pollution-intensive industries to developing nations. This hypothesis is supported by studies conducted by Frankel and Rose (2005) and Cole and Elliott (2005). However, Dinda (2004) counters that if this hypothesis were accurate, the influx of polluting industries would raise the income levels of host countries, prompting them to eventually impose stricter environmental regulations. Over time, this would result in a leveling of regulatory standards across countries, leaving no destination for polluting industries to relocate. In contrast, the Porter Hypothesis (PH), proposed by Porter and van der Linde (1995), argues that stringent environmental regulations serve as a catalyst for innovation. These regulations push firms to adopt cleaner and more efficient technologies, which in turn lower marginal costs and enhance productivity, ultimately making firms more competitive.

To evaluate the impact of environmental regulations on pollution, the OECD has developed the Environmental Policy Stringency (EPS) index. This index provides a country-specific, internationally comparable measure of the stringency of environmental regulations (Botta and Koźluk 2014) and covers 13 different policy instruments. The EPS index is divided into three equally weighted sub-indices, which classify policy instruments into market-based, non-market-based, and technology support policies (Kruse et al. 2022).

Numerous studies have explored the effectiveness of the Environmental Policy Stringency (EPS) index in reducing CO_2_ emissions. However, as noted by Albulescu et al. (2022), these studies often fail to reach consistent conclusions. It is important to note that CO_2_ emissions can be measured using two distinct methods. The most common is production-based accounting (PBA), which attributes emissions to the country where they are generated. The second method is consumption-based accounting (CBA) (Davis and Caldeira 2010; Wiedmann et al. 2015; Khan et al. 2020; Liddle 2018), which considers emissions embedded in international trade and the consumption patterns of a country’s citizens.

Most studies relying on PBA data (Albulescu et al. 2022; Ahmed and Ahmed 2018; Li et al. 2022; Sezgin et al. 2021; Wang et al. 2020, 2022; Yirong 2022) find that stringent environmental policies can lead to reductions in emissions. Sezgin et al. (2021) suggest that the effectiveness of EPS in reducing CO_2_ emissions increases with a country’s level of development. In contrast, Albulescu et al. (2022) find that EPS has a significant impact in reducing emissions in countries where per capita emissions are relatively low. Yirong (2022) offers a nuanced perspective: a linear model suggests stricter policies increase emissions, while a nonlinear model shows the opposite effect.

Additionally, the influence of EPS on CBA emissions is supported by Hassan et al. (2022), indicating that EPS can also trigger emissions through consumption patterns. Wolde-Rufael and Weldemeskel (2020) and Wolde-Rufael and Mulat-Weldemeskel (2021) argue that the impact of strict environmental regulations takes time to materialize. Finally, Guo et al. (2021) are the only researchers to analyze the effects of two types of EPS instruments on CO_2_ emissions. They find that both command-and-control measures and market-based environmental regulations contribute to reducing greenhouse gas emissions in OECD countries.

Our study aims to address two key research gaps in the existing literature. First, while many studies examine the impact of environmental regulations on CO_2_ emissions, few analyze the effects of specific, disaggregated regulatory instruments. Most research has focused on broad regulatory frameworks, neglecting the nuances of individual policy tools. Second, there is limited understanding of how the stringency of environmental regulations affects CO_2_ emissions across countries with varying levels of development and emissions.

By addressing these gaps, our study builds on and extends the findings of Albulescu et al. (2022) and Guo et al. (2021) in several important ways. First, we assess the impact of three distinct types of environmental policy instruments – market-based, non-market-based, and technology support policies – on CO_2_ emissions, providing new insights into the effectiveness of different regulatory strategies. Second, we incorporate both production-based accounting (PBA) and consumption-based accounting (CBA) to deliver a more comprehensive analysis of environmental regulations’ effects, especially in the context of global trade and emissions shifting to less regulated countries. Third, we employ a novel statistical method, the Method of Moments Quantile Regression (MMQR) proposed by Machado and Silva (2019), which allows us to capture cross-country heterogeneity and offer deeper insights into how environmental policy stringency (EPS) affects nations with different emission levels. Lastly, we introduce the Notre Dame Global Adaptation Initiative Index (ND-GAIN Index) as a control variable, exploring how a country’s vulnerability to climate change influences its environmental policies. By addressing these gaps, our study seeks to provide actionable policy recommendations to reduce CO_2_ emissions and contribute to global climate change mitigation efforts.

The study makes four key contributions to the existing literature.

First, it is the first to evaluate the impact of three distinct types of environmental regulation instruments on CO_2_ emission reductions. By doing so, we can identify which policy tools are most effective in curbing CO_2_ emissions, assess the costs associated with mitigation, and develop more targeted and efficient CO_2_ reduction strategies.

Second, we utilize both production-based accounting (PBA) and consumption-based accounting (CBA) to compare how environmental regulations affect CO_2_ reductions. This comprehensive analysis offers a clearer picture of the effectiveness of policy instruments, considering the impact of globalization, trade openness, and the outsourcing of production to countries with less stringent regulations (Malik and Lan 2016; Papież et al. 2022a). This approach is crucial to the ongoing debate on the trade-environment relationship, especially concerning the Pollution Haven Hypothesis (PHH), which links trade flows to regulatory differences across countries. The findings may either support the PHH or lend evidence to the alternative Porter Hypothesis (PH).

Third, while most studies rely on traditional panel regression models (such as the Generalized Method of Moments or Cross-Sectionally Augmented Autoregressive Distributed Lag models) to analyze the impact of environmental regulations on CO_2_ reductions (Wolde-Rufael and Weldemeskel 2020; Wang et al. 2020, 2022; Guo et al. 2021; Sezgin et al. 2021; Wolde-Rufael and Mulat-Weldemeskel 2021; Hassan et al. 2022; Li et al. 2022; Yirong 2022), our study introduces a novel approach. We apply the panel quantile regression model with fixed effects, as proposed by Machado and Silva (2019). This method allows us to examine the impact of environmental regulations across different quantiles, providing valuable insights into how policies affect countries with the highest and lowest CO_2_ emissions per capita.

Fourth, we incorporate the Notre Dame Global Adaptation Initiative (ND-GAIN) Index, which measures a country’s vulnerability to climate change and its capacity to adapt to its adverse effects. By including this index, we explore the extent to which climate vulnerability motivates governments to take action on CO_2_ reduction and climate protection. This also addresses ethical considerations surrounding the responsibility of less vulnerable nations to mitigate climate change for the global good and the well-being of future generations.

The remainder of the study is structured as follows: Section "Literature review" reviews the literature, Section "Data" introduces the data, and Section "Conclusions and policy implications" outlines the methods used. Section "Results and discussion" presents the empirical results and discussion, while the final section summarizes the conclusions and outlines policy implications.

## Literature review

Environmental pollution is undeniably a negative externality, and market mechanisms alone are insufficient to address environmental challenges. To counter this, many countries implement environmental laws and policies aimed at encouraging behavioral changes and reducing emissions (Zhang and Wen 2008; Albulescu et al. 2022). Numerous studies have evaluated the effectiveness of these measures in lowering emissions. In this review, we highlight the impact of stricter environmental policies on emission reductions and identify other key factors that influence emissions.

As noted in the Introduction, the implementation of environmental regulations, including carbon regulations and taxes, plays a critical role in driving CO_2_ reduction and is one of the primary tools in combating climate change. These regulations have been quantified and assessed through the Environmental Policy Stringency (EPS) index, which is composed of three key elements: market-based instruments, non-market-based instruments, and technology support policies (Kruse et al. 2022).

The literature presents mixed findings centered on two contrasting hypotheses. The first is the Pollution Haven Hypothesis (PHH), introduced by Copeland and Taylor (1994). The PHH suggests that stringent environmental regulations in developed countries lead to pollution being outsourced to developing countries, resulting in increased pollution in the latter. This hypothesis is supported by studies such as Frankel and Rose (2005) and Cole and Elliott (2005), but is contradicted by the findings of Dinda (2004).

The second hypothesis, known as the Porter Hypothesis (PH), was proposed by Porter and van der Linde (1995). It posits that strict environmental regulations can have a positive impact on eco-innovation and enhance both economic and environmental performance. While these regulations impose costs on producers and consumers, they also generate societal benefits in the form of a cleaner environment. New regulations may signal to companies that their resource use is inefficient, prompting technological improvements that can offset compliance costs (Ramanathan et al. 2017). By aiming to mitigate pollution, stringent environmental policies not only improve environmental quality but also encourage investment in eco-friendly technologies and discourage research and development in environmentally harmful technologies. While early empirical studies have provided inconclusive evidence on the validity of the Porter Hypothesis (Brännlund and Lundgren 2009), more recent research supports its claims (Wang et al. 2020; Chen et al. 2023).

Studies that directly assess the impact of environmental regulations on emissions yield inconclusive results. Albulescu et al. (2022) find that the EPS index significantly affects CO_2_ emissions, particularly in countries with relatively low per capita emissions. However, Yirong (2022) offers conflicting evidence: in the short term, a linear model indicates that an increase in EPS leads to higher CO_2_ emissions, while a non-linear model suggests the opposite. Hassan et al. (2022) support a positive and significant relationship between EPS and CO_2_ emissions in both the short and long term, though their analysis relies on consumption-based emissions (CBA) data.

Conversely, Wolde-Rufael and Weldemeskel (2020), as well as Wolde-Rufael and Mulat-Weldemeskel (2021), propose an inverted U-shaped relationship between EPS and CO_2_ emissions, implying that stringent environmental policies take time to reveal their effectiveness. This pattern holds for both production-based (PBA) and consumption-based (CBA) CO_2_ emissions. Additionally, Guo et al. (2021) analyze the impact of two types of EPS instruments on CO_2_ emissions, showing that both command-and-control and market-based environmental regulations contribute to greenhouse gas reductions in OECD countries.

The literature examining the impact of the stringency of environmental regulations on CO_2_ emissions spans various geographical contexts, including China (Ahmed and Ahmed 2018), OECD countries (Wang et al. 2020; Guo et al. 2021; Hassan et al. 2022; Li et al. 2022), BRICS countries (Wolde-Rufael and Mulat-Weldemeskel 2020; Wang et al. 2022), seven emerging economies (Wolde-Rufael and Mulat-Weldemeskel 2021), top-emitter economies (Yirong 2022), and both OECD and BRICS countries (Sezgin et al. 2021; Albulescu et al. 2022). Most studies rely on production-based emissions, with only a few employing consumption-based emissions data (Hassan et al. 2022; Wolde-Rufael and Mulat-Weldemeskel 2021). Various econometric approaches are used, including panel ARDL models (Wolde-Rufael and Mulat-Weldemeskel 2020; Hassan et al. 2022; Li et al. 2022; Wang et al. 2022; Yirong 2022), panel mean group models such as the Augmented Mean Group (AMG) (Wolde-Rufael and Mulat-Weldemeskel 2021; Li et al. 2022), and Common Correlated Effects Mean Group (CCEMG) (Li et al. 2022). The Generalized Method of Moments (GMM) is also frequently employed (Hassan et al. 2019; Wang et al. 2020; Guo et al. 2021), while Albulescu et al. (2022) use the fixed-effect quantile panel data approach introduced by Canay (2011).

One of the most significant determinants of emissions is a country’s economic activity and wealth. In this context, the Environmental Kuznets Curve (EKC), which suggests an inverted U-shaped relationship between economic growth and environmental degradation, is typically analyzed. However, the conclusions drawn from EKC studies remain ambiguous, as highlighted in review papers by Saqib and Benhmad (2021) and Sinha and Shahbaz (2019), due to variations in time frames and empirical approaches. In their analysis of EU countries, Frodyma et al. (2022) even point to the limitations of the parametric EKC model, suggesting that the relationship between economic growth and emissions may be more complex than the traditional EKC framework implies. Similarly, Li and Wei (2021), in their study of the Chinese economy, identify a significant non-linear relationship between carbon emissions and economic growth.

Another key area of research focuses on the concept of decoupling, which proposes that economic growth can continue while emissions decrease. Haberl et al. (2020) provide a comprehensive review of 835 articles on this topic and find that while long-term absolute decoupling is rare, some industrialized countries have successfully decoupled GDP growth from both production- and consumption-based CO_2_ emissions.

Overall, while economic activity is the primary driver of emissions, the relationship is complex and influenced by various factors, including policy interventions and technological advancements.

The impact of trade openness on emissions is examined through two key theoretical lenses. The first theory does not specify the direction of the relationship between trade openness and emissions, but highlights the potential effects of scale, technology, and composition (Ansari et al. 2020). The second theory, the Pollution Haven Hypothesis (PHH), suggests that differences in environmental standards across countries lead polluting companies to relocate their production to nations with weaker regulations, resulting in negative environmental impacts due to trade openness (Copeland and Taylor 2004).

Empirical studies show varying results on the relationship between trade openness and environmental pollution. For instance, Fang et al. (2019), Shahzad et al. (2017), and Dou et al. (2021) find evidence of negative environmental consequences from trade openness. Conversely, studies by Gozgor (2017), Zhang et al. (2017), and Shahbaz et al. (2013) report positive effects of trade openness on environmental quality. Wang and Zhang (2021) attribute these inconsistent findings to differences in environmental standards across countries, emphasizing the need for country-specific analyses. Additionally, Shahbaz et al. (2018) propose using a broader measure of globalization, such as the Globalization Index (Dreher 2006), which encompasses not only economic but also social and political globalization. Our approach, which adopts this index, allows for a more comprehensive analysis of how trade openness impacts the environment in countries with varying levels of emissions.

Climate vulnerability reflects the extent to which people and ecosystems are affected by climate change. Previous studies have examined the negative impact of climate vulnerability on both microeconomic and macroeconomic performance (Huang et al. 2018; Kling et al. 2021; Kahn et al. 2021), suggesting that it may lead to reduced economic activity, which in turn influences greenhouse gas (GHG) emissions. However, some researchers argue that climate vulnerability could also stimulate green investment. For example, Marshall et al. (2021) observe greater inflows into green mutual funds in climate-vulnerable regions of the U.S, while Wen et al. (2023) – using panel data from 107 countries – find that climate vulnerability actually decreases investment in both climate change mitigation and adaptation technologies. Our study aims to assess the direct impact of climate vulnerability on CO_2_ emissions. Following recent studies (Kling et al. 2021; Wen et al. 2023), we use the ND-GAIN index as an indicator of climate vulnerability.

While previous research provides a consistent explanation of how environmental regulation stringency affects emissions, this study seeks to fill a gap by examining how specific components of the EPS index can effectively reduce CO_2_ emissions. In addition, we explore two widely recognized determinants of CO_2_ emissions and assess the impact of the ND-GAIN index, which has not yet been studied in this context. Moreover, we compare the effects of the EPS index on both production-based (PBA) and consumption-based (CBA) emissions. Notably, we employ the Method of Moments Quantile Regression (MMQR) procedure, as proposed by Machado and Silva (2019), which enables a comparison of the environmental impact of determinants across both low and high emitters.

## Data

The analysis covers 38 countries in the period between 1995 and 2020, using annual data. The list of countries is provided in Table 1.

**Table 1:** List of countries

List of countries	
Australia, Austria, Belgium, Brazil, Canada, Chile, China, Czechia, Denmark, Estonia, Finland, France, Germany, Greece, Hungary, India, Ireland, Israel, Italy, Japan, Luxembourg, Mexico, Netherlands, New Zealand, Norway, Poland, Portugal, Russia, Slovakia, Slovenia, South Africa, Spain, Sweden, Switzerland, Turkey, United Kingdom, United States, South Korea	

The variables used to assess the impact of environmental regulations on CO_2_ emissions are listed in Table 2. The dependent variables are two measures of CO_2_ emissions: production-based emissions per capita (PBApc) and consumption-based emissions per capita (CBApc), both expressed in metric tonnes per capita. PBA emissions refer to emissions generated from the production of goods and services, assigned to the country where the emissions occur. This aligns with the definition used in the System of National Accounts (SNA 1993), as referenced by Karstensen et al. (2018). In contrast, CBA emissions are calculated by subtracting the emissions embedded in exports from PBA emissions and adding the emissions embedded in imports. This approach captures the international flow of emissions, accounting for emissions generated in one country but consumed in another (Liddle 2018; Hasanov et al. 2018; Karakaya et al. 2019; Khan et al. 2020). Both CO_2_ emission indicators are sourced from the Our World in Data database.

**Table 2:** Data description

Symbol	Variable	Unit	Data source
PBApc	Production-based emissions per capita	metric tonnes per capita	Our World in Data
CBApc	Consumption-based emissions per capita	metric tonnes per capita	Our World in Data
EPS	Environmental policy stringency index	Index	OECD database
Nm_EPS	Non-market EPS, sub-index of EPS	Index	OECD database
M_EPS	Market EPS, sub-index of EPS	Index	OECD database
Ts_EPS	Technology support EPS, sub-index of EPS	Index	OECD database
GDPpc	Gross domestic product per capita	at constant prices 2015 US dollar	WDI
KOF	KOF Index of globalization	Index	KOF Swiss Economic Institute
Gain	ND-GAIN Country Index	Index	Notre Dame Global Adaptation Initiative

**Fig. 1:**
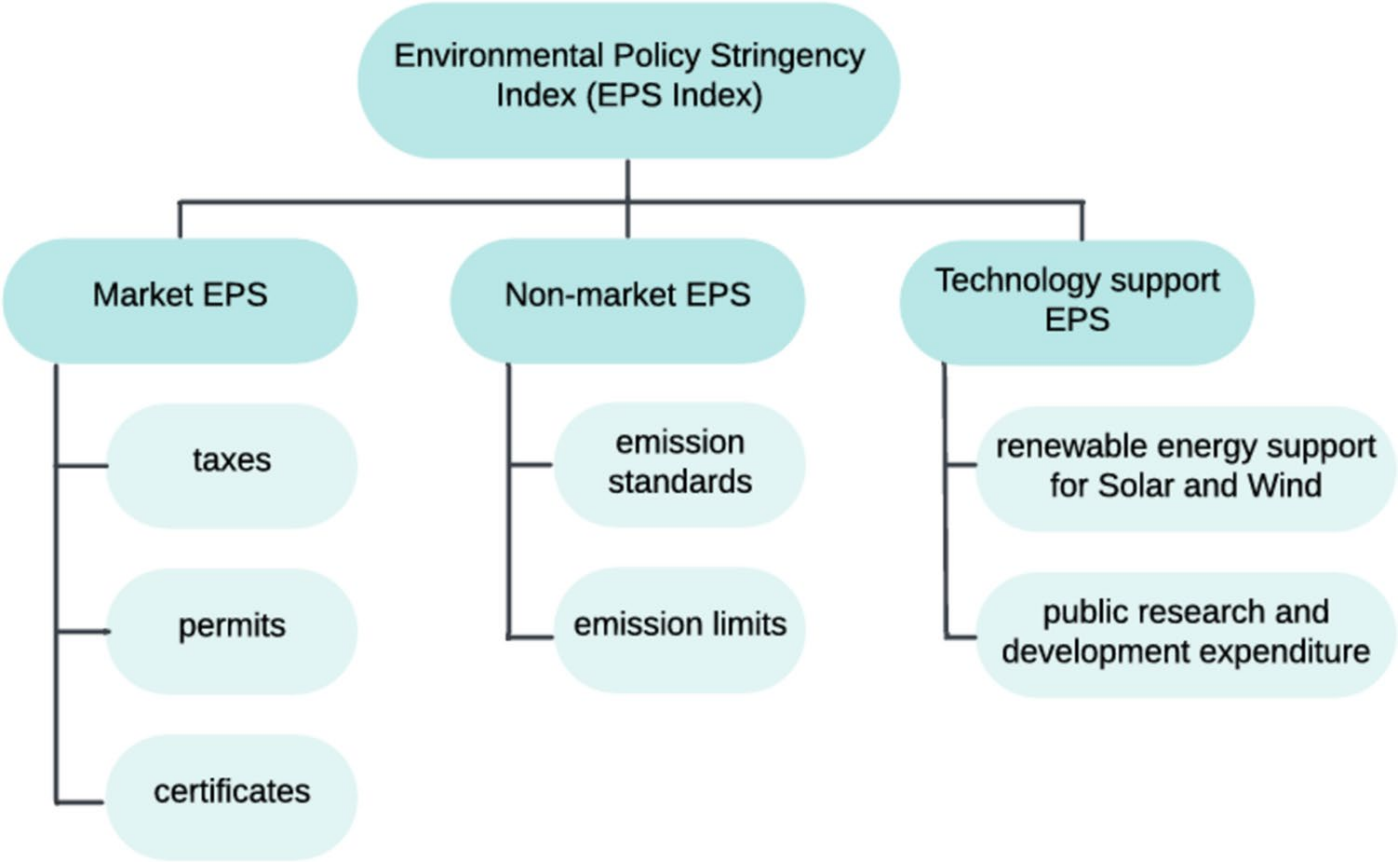
Components of the EPS Index, the scheme adapted from (Kruse et al. 2022)

Next, the EPS index and its sub-indices are analyzed. The EPS index evaluates the extent to which environmental policies impose direct or indirect costs on activities that contribute to pollution or are environmentally harmful. The index ranges from zero to six, with a score of six representing the highest level of stringency in environmental regulation. The previous iteration of the EPS index included only two sub-indices: market-based and non-market-based policies. However, it has been updated to incorporate an additional sub-index – technology support by Kruse et al. (2022). Figure 1 illustrates the structure of the revised EPS index, which comprises three equally weighted sub-indices. The non-market EPS sub-index (Nm_EPS) addresses performance standards by mandating emission limits and regulations. The market EPS sub-index (M_EPS) evaluates policies that put a price on pollution, such as taxes, permits, and certificates. The technology support EPS sub-index (Ts_EPS) refers to support for innovation in clean technologies, including public research and development expenditures as well as renewable energy initiatives for solar and wind technologies. All EPS indices and their sub-indices are sourced from the OECD database.

Additionally, three control variables are considered in the analysis. Gross domestic product per capita (GDPpc), measured in constant 2015 U.S. dollars, is obtained from the World Development Indicators (WDI) database. Globalization is assessed using economic, social, and political indices based on the framework provided by Dreher (2006). The KOF Globalization Index, developed by the KOF Swiss Economic Institute, measures globalization on a scale from zero to one hundred, with higher values indicating a greater degree of global integration. The Notre Dame Global Adaptation Index (ND-GAIN), proposed by the University of Notre Dame, evaluates a country’s current vulnerability to climate disruptions and its preparedness to utilize investments from both the private and public sectors for adaptive measures. The ND-GAIN index provides insight into a country’s climate-related vulnerabilities and its capacity for response and adaptation to climate change. Its values range from zero to one hundred, with higher scores reflecting a country’s greater ability to leverage investments for effective adaptation actions. The ND-GAIN index is sourced from the Notre Dame Global Adaptation Initiative Database.

**Fig. 2:**
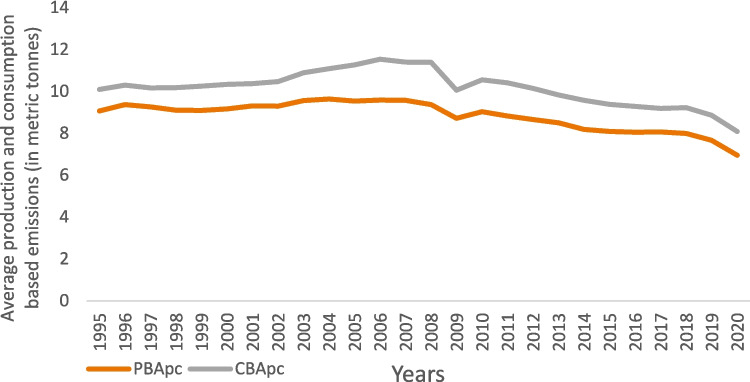
Average CO_2_ emissions per capita based on production and consumption for the period 1995 to 2020

Descriptive statistics for all countries in 1995 and 2020 are provided in Appendix Table 10. The average production-based emissions per capita (PBApc) are significantly lower than the average consumption-based emissions per capita (CBApc), with both metrics showing a decline over the analyzed period (Fig. 2). Specifically, the average PBApc emissions were 9.07 in 1995, compared to 6.95 in 2020, while CBApc emissions decreased from 10.10 to 8.09 over the same timeframe. This indicates a negative trend in emissions over time. Notably, countries such as Australia, Canada, and Russia exhibit lower CBApc emissions than PBApc emissions (see Figure 5 in the Appendix). Additionally, the variability in both CBApc and PBApc emissions is lower in 2020 than it was in 1995.

**Fig. 3:**
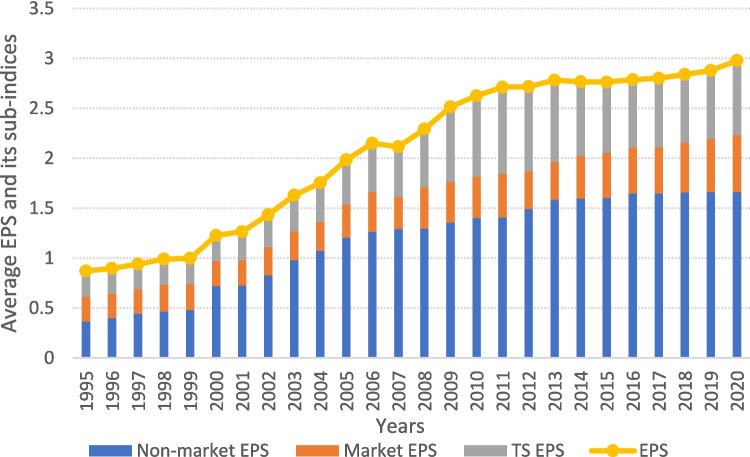
The average EPS index and its sub-indices between 1995 and 2020

The mean values of the EPS index and its sub-indices are significantly lower in 1995 than in 2020, indicating that policy stringency was initially low but gradually increased to a moderate level by 2020. Only several countries, including Denmark, Finland, France, Italy, Japan, Luxembourg, and Switzerland, maintain an EPS index above 4 during the analyzed period. The rise in the EPS index is primarily attributed to an increase in the non-market EPS sub-index, which reflects the implementation of emission limits and standards (Fig. 3).

Furthermore, an upward trend in the EPS index and its sub-indices is evident over time, regardless of the CO_2_ measurement employed. The minimum EPS index is recorded in Chile and Slovenia (0.00) in 1995, while the maximum in France in 2020 (4.89). The mean values of the EPS index, Nm_EPS, M_EPS, and Ts_EPS are 0.87, 1.09, 0.75, and 0.77 in 1995, respectively, and increase to 2.97, 4.96, 1.74, and 2.22 in 2020. Environmental regulations tend to be more effective in countries with relatively low per capita emissions compared to those with higher pollution levels.

The average KOF Globalization Index rises from 67.75 in 1995 to 80.91 in 2020, suggesting an increase in globalization. The highest KOF index is observed in the Netherlands in 2020 (90.61), while the lowest is recorded in India in 1995 (39.49). Similarly, the mean value of the Notre Dame Global Adaptation Index (ND-GAIN) is higher in 2020 than in 1995, indicating an enhanced ability of countries to leverage investments and effectively translate them into adaptation actions. The minimum ND-GAIN index is found in India in 2015 (38.88), while the maximum in Norway in 2015 (77.32).

## Methodology

**Fig. 4:**
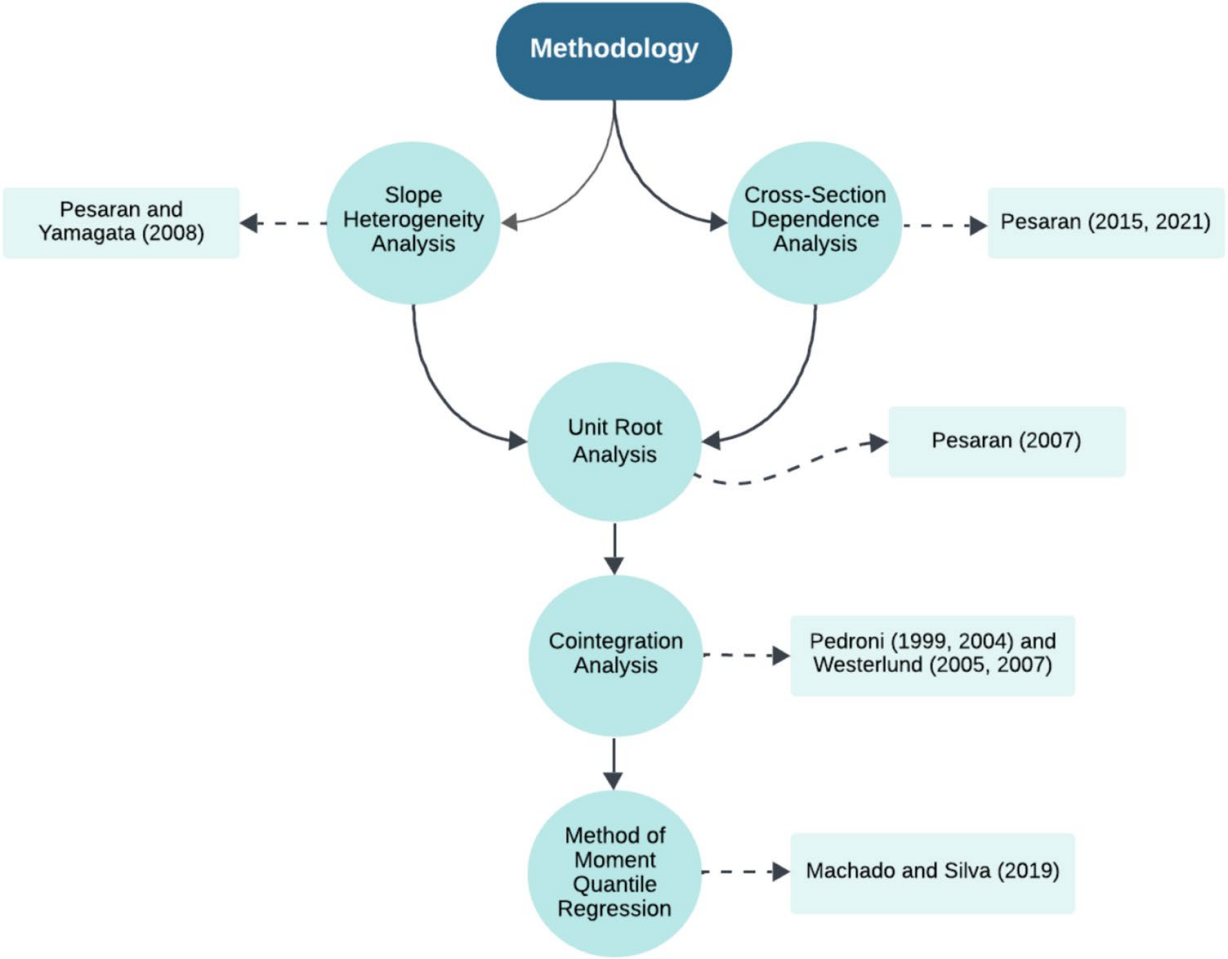
The methodological structure of the study

**Fig. 5:**
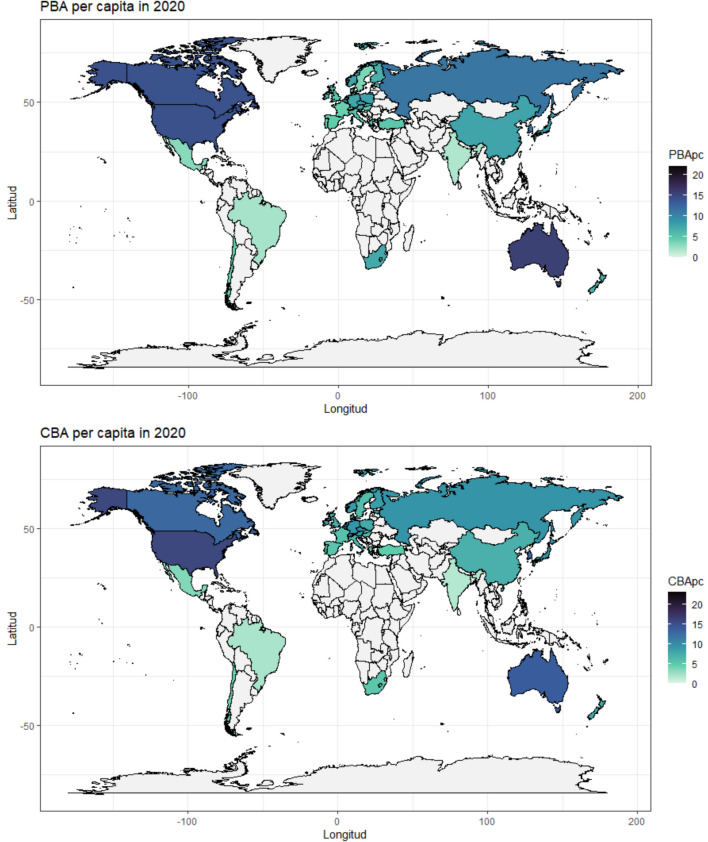
PBA and CBA emissions per capita in 2020

The methodology of the study is detailed in the following section, with a visual representation of the process provided in Fig. 4.

### Preliminary analysis

Before estimating the panel quantile regression, cross-sectional dependence, stationarity and the long-run relationship among the variables are tested.

The cross-sectional dependence (CD) tests of Pesaran (2021, 2015) are used to assess cross-sectional dependence. The null hypotheses for these tests are that there is no (Pesaran 2021) and weak (Pesaran 2015) cross-sectional dependence. The test statistics are as follows:

1 $$CD={\left(\frac{1}{N(N-1)}\right)}^{1/2}\sum\nolimits_{i=1}^{N-1}\sum\nolimits_{j=i+1}^{N}\left(T{\widehat{\rho }}_{ij}^{2}-1\right)$$

where ${\widehat{\rho }}_{ij}^{2}$ is the correlation.

Homogeneity is evaluated using the Pesaran and Yamagata (2008) test. The null hypothesis posits that slope homogeneity exists across all individuals being compared, while the alternative hypothesis indicates the presence of slope heterogeneity in the non-zero portion of the pairwise slopes. The test statistics are as follows:

2 $${\widetilde{\Delta }}_{SH}={\left(N\right)}^\frac{1}{2}{\left(2k\right)}^{-\frac{1}{2}}\left(\frac{1}{N}\widetilde{S}-k\right)$$

3 $$\widetilde{S}=\sum\nolimits_{i=1}^{N}\left({\beta }_{i}-{\beta }_{WFE}\right)\frac{{{X}{\prime}}_{i}{M}_{\tau }{X}_{i}}{{\sigma }_{i}^{2}}\left({\beta }_{i}-{\beta }_{WFE}\right)$$

4 $${\widetilde{\Delta }}_{Adjusted- SH}={\left(N\right)}^\frac{1}{2}{\left(\frac{2k\left(T-k-1\right)}{T+1}\right)}^{-\frac{1}{2}}\left(\frac{1}{N}\widetilde{S}-2k\right),$$

where ${\beta }_{WFE}$—the coefficient of the pooled weighted fixed effects estimator; ${\beta }_{i}$—the coefficient of the pooled OLS $; k$—the number of control variables, and $\widetilde{S}$—the Swamy’s test statistic.

The cross-section augmented IPS (CIPS) panel unit test (Pesaran 2007) assesses the stationarity of the data. The null hypothesis for this test states that a unit root exists in the data, and the statistic is calculated using the following formula:

5 $$CIPS\left(N,T\right)=\frac{1}{N}\sum\nolimits_{i=1}^{N}{t}_{i}\left(N,T\right)$$

where ${t}_{i}\left(N,T\right)$—the cross-sectional augmented Dickey–Fuller (CADF) statistic for the *ith* cross-sectional unit indicated by the t-ratio of y_i, t−1_ coefficient in the CADF regression.

Cointegration relationships are tested using methodologies developed by Pedroni (1999, 2004) and Westerlund (2005, 2007). Pedroni’s test accommodates panel-specific cointegrating vectors, while Westerlund’s approach addresses cross-sectional dependence and heterogeneity. Westerlund’s (2007) cointegration test relies on four different test statistics: two group mean statistics (Gt and Ga) and two panel statistics (Pt and Pa). The null hypothesis asserts that there is no cointegration for the group or panel test statistics. The alternative hypothesis posits that at least one cross-sectional unit is cointegrated in the group mean statistic, while the panel as a whole is cointegrated in the panel statistic.

### Method of moments quantile regression

To analyze the determinants of CO_2_ emissions, the study employs a novel Method of Moments Quantile Regression (MMQR) technique recently proposed by Machado and Silva (2019). While the first-generation quantile regression methods introduced by Koenker and Bassett (1978) exist, the study employs the second-generation MMQR approach to avoid the limitations of the earlier methods. Specifically, first-generation techniques do not adequately account for unobserved heterogeneity among data points and panel cross-sections, which can lead to inefficient and potentially misleading estimations, as highlighted by Zhu et al. (2016).

The MMQR model enables capturing variations in CO_2_ emissions per capita and several covariates, including the components of the EPS index across different quantiles among OECD and BRICS countries. This approach effectively addresses heterogeneity and distributional differences. Moreover, this model can account for covariance effects across the entire distribution, a capability that first-generation quantile regression techniques do not possess. Furthermore, MMQR can identify asymmetries in covariates based on their positions and mitigate the influence of their endogenous properties, as noted by Anwar et al. (2021). Also, in a nonlinear context, estimates obtained through MMQR are more robust, reliable, comparable, and reproducible. Building on the framework established by Machado and Silva (2019), the fixed-effects panel quantile model can be expressed as follows:

6 $${Q}_{{Y}_{i,t}}\left(\tau |{X}_{i,t}\right)=\left({\gamma }_{i}+{\beta }_{i}(q\left(\tau \right)\right)+{X^{\prime}}_{i,t}\alpha +{Q}_{it}^{\prime}{\delta }_{q}\left(\tau \right), i=1,..,N, t=1,..,T$$

where ${Y}_{i,t}$ represents the PBA and CBA emissions per capita (PBApc, CBApc); ${X^{\prime}}_{i,t}$ represents the dependent variables i.e. EPS indices, GDP per capita, KOF index, ND-GAIN Country Index (Gain); $\gamma , \alpha , \beta ,$ and $\delta$ are parameters that have to be estimated. The left-hand side of the Eq. (6) denotes the conditional quantile distribution of the endogenous variable. Unlike other LS-fixed effects techniques, there is no intercept term for individual effects. The variables are expected to be constant over time, which means that the variation among the units is subject to change.

## Results and discussion

### Model specification

Our analysis is grounded in the framework of the Environmental Kuznets Curve (EKC), which we enhance by incorporating the Environmental Policy Stringency (EPS) index alongside other control variables. The primary aim is not to test the validity of the EKC hypothesis but to evaluate the impact of environmental policy stringency, including its individual components, on the reduction of CO₂ emissions. This methodological approach has been adopted in several studies, such as those by Wolde-Rufael and Mulat-Weldemeskel (2021) and Zhang et al. (2017).

To investigate the influence of the EPS index on CO₂ emissions, we consider four types of baseline models derived from Eq. (6) and based on the EKC framework. Two of these models utilize the aggregated EPS index (PBA_MODEL 0 and CBA_Model 0), while the other two focus on the sub-indices of the EPS index (PBA_MODEL 1 and CBA_Model 1). We separately examine two measures of CO₂ emissions: PBA and CBA emissions. The specifications of these models are presented in Table 3:

**Table 3:** List of baseline models

PBA_Model 0	${lnPBApc}_{it}={\alpha }_{0}+{\alpha }_{1}{lnGDPpc}_{it}+{{\alpha }_{2}{\left({lnGDPpc}_{it}\right)}^{2}+\alpha }_{3}{lnEPS}_{it}+{\varepsilon }_{it}$
PBA_Model 1	${lnPBApc}_{it}={\alpha }_{0}+{\alpha }_{1}{lnGDPpc}_{it}+{{\alpha }_{2}{\left({lnGDPpc}_{it}\right)}^{2}+\alpha }_{3}{lnNm\_EPS}_{it}+{\alpha }_{4}{lnM\_EPS}_{it}+{\alpha }_{5}{lnTs\_EPS}_{it}+{\varepsilon }_{it}$
CBA_Model 0	${lnCBApc}_{it}={\alpha }_{0}+{\alpha }_{1}{lnGDPpc}_{it}{+\alpha }_{2}{\left({lnGDPpc}_{it}\right)}^{2}+{\alpha }_{3}{lnEPS}_{it}++{\varepsilon }_{it}$
CBA_Model 1	${lnCBApc}_{it}={\alpha }_{0}+{\alpha }_{1}{lnGDPpc}_{it}+{\alpha }_{2}{\left({lnGDPpc}_{it}\right)}^{2}+{\alpha }_{3}{lnNm\_EPS}_{it}+{\alpha }_{4}{lnM\_EPS}_{it}+{\alpha }_{5}{lnTs\_EPS}_{it}++{\varepsilon }_{it}$

where ${lnPBApc}_{it}$ and ${lnCBApc}_{it}$ are the logarithms of PBA and CBA emissions per capita, ${lnGDPpc}_{it}$ is the logarithm of *GDP* per capita, *lnEPS* is the logarithm of the EPS index, ${lnNm\_EPS}_{it}, {lnM\_EPS}_{it},{lnTs\_EPS}_{it}$ correspond to the components of ESP, namely non-market EPS, market EPS and technology support EPS,${\alpha }_{i}$ are model parameters, subscripts *i* and *t*, correspond to country *i* and year *t*.

In addition, we incorporate two control variables into the baseline model, resulting in six additional models. The first two models include both control variables: the logarithm of the ND-GAIN index (lnGain) and the logarithm of the KOF index (lnKOF) for both CO₂ measures designated as PBA_Model 0_GAIN_KOF or CBA_Model_0_GAIN_KOF. The subsequent four models incorporate three EPS components along with one control variable,^1^ referred to as (i.e. PBA_Model 1_GAIN /CBA_Model 1_GAIN or PBA_Model 1_KOF/CBA_Model 1_KOF). The parameters of each model are estimated across various quantiles, allowing us to identify different determinants for countries with varying levels of emissions.

### Results of diagnostic tests

First, we conduct Pesaran’s cross-sectional dependence (CD) test (Pesaran 2021, 2015) and the slope homogeneity test (Pesaran and Yamagata 2008). These tests are crucial since the panel unit root test developed by Pesaran (2007) presupposes cross-sectional dependence within the panel data. The results of the CD tests (Pesaran 2021, 2015), the homogeneity of slopes across countries test of Pesaran and Yamagata (2008), and the unit root test of Pesaran (2007) are reported in Tables 11, 12, 13, and 14 in the Appendix. The CD test indicates that all variables are affected by cross-sectional dependence, and the slope homogeneity test accepts the alternative hypothesis of heterogeneous slopes at the 1% significance level for all models (i.e., the baseline models and models with control variables). According to the CIPS test results, all variables are I(1). The results of the cointegration tests for the baseline models are presented in Appendix Table 15.^2^ Both the Pedroni (2004, 1999) and Westerlund (2005) tests reveal that all variables are cointegrated in all models. The results of the group (Gt or Ga) and panel (Pt or Pa) statistics of the Westerlund (2007) test indicate stable long-run cointegration relationships in all four models at the 10% significance level.

### Effects of environmental regulation stringency on CO2 emissions in the baseline models

Tables 4 and 5 present the results regarding the impact of the EPS index on PBA and CBA emissions in the baseline models based on the EKC. Across all quantiles analyzed, the parameter for the EPS index is consistently significant and negative, indicating that more stringent environmental regulations lead to a reduction in emissions, regardless of whether they are measured as PBA or CBA.

**Table 4:** PBA_Model 0 (Quantile Regression)

VARIABLES	location	scale	qtile_10	qtile_30	qtile_50	qtile_70	qtile_90
lnGDPpc	3.470***	−0.397***	4.077***	3.746***	3.409***	3.138***	2.930***
	(18.54)	(−4.112)	(15.77)	(18.21)	(18.05)	(16.21)	(13.83)
lnGDPpc2	−0.167***	0.0221***	−0.200***	−0.182***	−0.163***	−0.148***	−0.136***
	(−15.01)	(3.838)	(−13.38)	(−15.15)	(−14.51)	(−12.62)	(−10.52)
**lnEPS**	−0.224***	0.0120	−0.243***	−0.233***	−0.223***	−0.214***	−0.208***
	(−10.08)	(0.935)	(−7.286)	(−9.049)	(−10.18)	(−9.528)	(−8.215)
Constant	−15.62***	1.815***	−18.40***	−16.88***	−15.34***	−14.11***	−13.15***
	(−19.57)	(4.424)	(−16.01)	(−18.70)	(−19.22)	(−17.79)	(−15.44)

z-statistics in parentheses ***p < 0.01, **p < 0.05, *p < 0.1

**Table 5:** CBA_Model 0 (Quantile Regression)

VARIABLES	location	scale	qtile_10	qtile_30	qtile_50	qtile_70	qtile_90
lnGDPpc	3.048***	−0.448***	3.753***	3.382***	3.014***	2.704***	2.407***
	(15.49)	(−4.416)	(15.07)	(16.31)	(14.92)	(12.59)	(9.711)
lnGDPpc2	−0.142***	0.024***	−0.180***	−0.160***	−0.140***	−0.124***	−0.108***
	(−12.03)	(3.945)	(−12.44)	(−13.12)	(−11.58)	(−9.423)	(−7.044)
lnEPS	−0.161***	0.0298**	−0.208***	−0.183***	−0.159***	−0.138***	−0.119***
	(−6.384)	(2.119)	(−5.797)	(−6.437)	(−6.289)	(−5.255)	(−3.975)
Constant	−13.80***	2.135***	−17.15***	−15.39***	−13.63***	−12.16***	−10.75***
	(−16.79)	(4.995)	(−15.61)	(−17.16)	(−16.18)	(−14.01)	(−10.93)

z-statistics in parentheses ***p < 0.01, **p < 0.05, *p < 0.1

Two key observations arise from these findings. First, the EPS index exerts a more substantial influence on reducing PBA emissions compared to CBA emissions, as evidenced by the smaller (more negative) parameters associated with PBA. This outcome aligns with expectations, given that these policies primarily target domestic market conditions rather than external markets, a point also noted by Papież et al. (2022a). Second, the impact of the EPS index varies based on the level of emissions: it is weaker in countries with higher per capita emissions compared to those with lower emissions. This phenomenon may be attributed to the fact that high-emission countries, such as Australia, the United States, Canada, and Russia, are significant producers and exporters of fossil fuels. Since CO_2_ emissions are closely linked to production levels, it becomes more challenging to curtail emissions in these countries, even with relevant policies in place.

The findings of our study align with those of Albulescu et al. (2022), who demonstrate that increasing environmental stringency leads to a reduction in CO_2_ emissions among 32 OECD countries in the period between 1990 and 2015. Their research also reveals that the impact of environmental stringency is more pronounced in countries with relatively low per capita emissions. Similar results are obtained by Albulescu et al. (2020) in the EU countries, Zhao et al. (2015) in China, and Guo et al. (2021) across 27 OECD countries. However, Niedertscheider et al. (2018) report opposing results in Australia. Moreover, Wolde-Rufael and Weldemeskel (2020) observe different outcomes in the BRICS countries, while Wolde-Rufael and Mulat-Weldemeskel (2021) note similar findings in seven developing nations for both measures of CO_2_ emissions. They find that although stringent environmental policies may not yield immediate environmental benefits, they become effective after reaching a certain threshold. Their conclusion emphasizes that the effectiveness of stringent environmental policies requires time to manifest.

Tables 6 and 7 present the results of disaggregating the EPS index into three components: non-market-based (lnNm_EPS), market-based (lnM_EPS), and technology support (lnTs_EPS) instruments. From this analysis, three key conclusions can be drawn regarding the effectiveness of these different environmental policy instruments.

**Table 6:** PBA_Model 1 (Quantile Regression)

VARIABLES	location	scale	qtile_10	qtile_30	qtile_50	qtile_70	qtile_90
lnGDPpc	3.355***	−0.373***	3.924***	3.620***	3.307***	3.045***	2.849***
	(19.32)	(−3.687)	(15.99)	(18.76)	(18.89)	(16.32)	(13.36)
lnGDPpc2	−0.159***	0.021***	−0.191***	−0.174***	−0.157***	−0.142***	−0.131***
	(−15.42)	(3.565)	(−13.67)	(−15.59)	(−14.94)	(−12.45)	(−10.000)
lnNm_EPS	−0.074***	0.016*	−0.099***	−0.086***	−0.073***	−0.061***	−0.052***
	(−4.476)	(1.909)	(−4.116)	(−4.478)	(−4.420)	(−3.791)	(−3.026)
lnM_EPS	−0.186***	0.0101	−0.201***	−0.193***	−0.185***	−0.177***	−0.172***
	(−6.125)	(0.673)	(−4.644)	(−5.544)	(−6.182)	(−6.047)	(−5.483)
lnTs_EPS	−0.055***	−0.024***	−0.018	−0.037**	−0.058***	−0.075***	−0.088***
	(−3.577)	(−2.980)	(−0.787)	(−2.133)	(−3.785)	(−4.938)	(−5.251)
Constant	−15.170***	1.688***	−17.750***	−16.370***	−14.960***	−13.770***	−12.880***
	(−20.45)	(3.813)	(−15.91)	(−19.02)	(−20.22)	(−18.12)	(−15.01)

z-statistics in parentheses ***p < 0.01, **p < 0.05, *p < 0.1

**Table 7:** CBA_Model 1 (Quantile Regression)

VARIABLES	location	scale	qtile_10	qtile_30	qtile_50	qtile_70	qtile_90
lnGDPpc	2.963***	−0.399***	3.596***	3.261***	2.918***	2.657***	2.407***
	(15.34)	(−3.490)	(13.75)	(15.55)	(14.81)	(12.38)	(9.471)
lnGDPpc2	−0.137***	0.0213***	−0.171***	−0.153***	−0.134***	−0.120***	−0.107***
	(−11.56)	(3.063)	(−11.00)	(−12.16)	(−11.12)	(−8.994)	(−6.719)
lnNm_EPS	−0.060***	0.034***	−0.114***	−0.086***	−0.056***	−0.034*	−0.013
	(−3.306)	(3.647)	(−4.417)	(−4.090)	(−3.105)	(−1.880)	(−0.637)
lnM_EPS	−0.127***	0.007	−0.139***	−0.133***	−0.126***	−0.121***	−0.117***
	(−4.026)	(0.491)	(−3.384)	(−3.863)	(−4.004)	(−3.706)	(−3.198)
lnTs_EPS	−0.031*	−0.027***	0.012	−0.011	−0.034*	−0.051***	−0.068***
	(−1.710)	(−2.989)	(0.450)	(−0.514)	(−1.878)	(−2.971)	(−3.613)
Constant	−13.460***	1.913***	−16.500***	−14.890***	−13.240***	−11.990***	−10.790***
	(−17.11)	(4.086)	(−14.70)	(−16.81)	(−16.60)	(−14.23)	(−10.94)

z-statistics in parentheses ***p < 0.01, **p < 0.05, *p < 0.1

First, the tightening of market-based instruments – those that put a price on pollution – demonstrates the most significant impact on emission reductions. The coefficients for these instruments range from −0.172 to −0.201 for PBA and −0.117 to −0.139 for CBA emissions. This indicates that increasing the stringency of renewable energy certificates, CO_2_ trading schemes, and taxes on nitrogen oxides (NOx), sulfur oxides (SOx), and CO_2_ leads to a notable reduction in CO_2_ emissions. In contrast, non-market-based policies that set emission limits and standards for pollutants such as NOx, SOx, and particulate matter from new coal-fired power plants exhibit coefficients ranging from −0.0524 to −0.0986 for PBA and −0.0128 to −0.114 for CBA. These findings suggest that while performance standards have a positive effect on emission reductions, their impact is comparatively weaker than that of market-based instruments. Finally, technology support instruments, which promote innovation in clean technologies – particularly for solar and wind energy – yield the least impact on emissions. The parameters for these instruments range from −0.0176 to −0.0879 for PBA and from 0.0116 to −0.0678 for CBA, indicating a generally positive but limited influence on environmental outcomes. Our findings align with those of Guo et al. (2021), who confirm that both command-and-control regulations and market-based environmental policies positively affect GHG emission reductions in OECD countries.

Second, increased stringency in environmental regulations results in more significant reductions in PBA emissions compared to CBA emissions. This trend holds true for the individual components of the EPS index as well. This outcome aligns with expectations, as these policies are primarily designed to target emission reductions within the countries where they are implemented. This phenomenon is also reported in the study by Papież et al. (2022a). Furthermore, the smaller impact of environmental policy stringency observed on CBA emissions compared to PBA emissions supports the validity of the Pollution Haven Hypothesis (PHH). Notably, only countries with the lowest CO_2_ emissions experience a greater reduction in CBA emissions than in PBA emissions.

Third, the effectiveness of two types of policies – market-based (lnM_EPS) and non-market-based (lnNm_EPS) – tends to diminish in countries with higher per capita CO_2_ emissions, particularly for larger quantiles. The opposite relationship is observed for technology support instruments (lnTs_EPS), indicating that countries with the highest per capita emissions have the greatest potential for reducing emissions, provided that their policies emphasize these instruments. This significant finding underscores the necessity for tailored policy tools suited to countries with varying emission levels and highlights the importance of investing in research and development, especially in high-emission countries.

### Robustness check – the impact of environmental regulation stringency on CO2 emissions in the models with control variables

Tables 8 and 9 present the regression coefficients for models that include two control variables: globalization (lnKOF) and a country’s vulnerability to climate change (lnGain), excluding economic growth (lnGDP) and its square. These results yield several important observations.

**Table 8:** PBA_Model 0_GAIN_KOF (Quantile Regression)

VARIABLES	location	scale	qtile_10	qtile_30	qtile_50	qtile_70	qtile_90
LnGDPpc	3.617***	−0.425***	4.265***	3.913***	3.556***	3.262***	3.028***
	(19.20)	(−4.240)	(16.67)	(19.15)	(18.57)	(16.27)	(13.48)
LnGDPpc2	−0.172***	0.026***	−0.211***	−0.189***	−0.168***	−0.150***	−0.136***
	(−15.21)	(4.365)	(−14.11)	(−15.73)	(−14.57)	(−12.35)	(−9.970)
LnEPS	−0.196***	0.020	−0.226***	−0.210***	−0.193***	−0.179***	−0.168***
	(−8.540)	(1.497)	(−6.368)	(−7.764)	(−8.589)	(−7.941)	(−6.680)
LnKOF	−0.287***	−0.008	−0.275**	−0.281***	−0.288***	−0.293***	−0.297***
	(−3.166)	(−0.151)	(−2.075)	(−2.731)	(−3.217)	(−3.169)	(−2.849)
LnGain	−0.0491	−0.281***	0.379**	0.146	−0.0894	−0.283**	−0.438***
	(−0.380)	(−4.162)	(2.233)	(1.063)	(−0.678)	(−2.023)	(−2.768)
Constant	−15.18***	2.914***	−19.62***	−17.21***	−14.76***	−12.75***	−11.14***
	(−15.70)	(6.013)	(−14.44)	(−15.90)	(−15.01)	(−13.03)	(−10.54)

z-statistics in parentheses ***p < 0.01, **p < 0.05, *p< 0.1.

**Table 9:** CBA_Model 0_GAIN_KOF (Quantile Regression)

VARIABLES	location	scale	qtile_10	qtile_30	qtile_50	qtile_70	qtile_90
LnGDPpc	3.284***	−0.416***	3.941***	3.578***	3.233***	2.946***	2.694***
	(15.80)	(−3.827)	(15.09)	(16.35)	(15.27)	(12.92)	(10.12)
LnGDPpc2	−0.148***	0.0245***	−0.187***	−0.166***	−0.145***	−0.128***	−0.113***
	(−11.64)	(3.795)	(−11.70)	(−12.29)	(−11.23)	(−9.294)	(−7.094)
LnEPS	−0.100***	0.0244*	−0.139***	−0.117***	−0.0972***	−0.0804***	−0.0656**
	(−3.806)	(1.731)	(−3.554)	(−3.840)	(−3.737)	(−3.125)	(−2.306)
LnKOF	−0.514***	−0.0186	−0.485***	−0.501***	−0.516***	−0.529***	−0.540***
	(−5.153)	(−0.352)	(−4.011)	(−4.948)	(−5.121)	(−4.663)	(−4.044)
LnGain	−0.300*	−0.150*	−0.0643	−0.195	−0.319**	−0.422**	−0.513***
	(−1.872)	(−1.802)	(−0.296)	(−1.100)	(−2.002)	(−2.526)	(−2.682)
Constant	−12.14***	2.458***	−16.02***	−13.87***	−11.84***	−10.14***	−8.650***
	(−11.17)	(4.361)	(−10.40)	(−11.13)	(−10.90)	(−9.392)	(−7.111)

z-statistics in parentheses ***p < 0.01, **p < 0.05, *p < 0.1

First, they reaffirm the conclusions drawn from Tables 4 and 5 regarding the strength and direction of the impact of the EPS index on CO_2_ emissions across countries with varying emission levels. Specifically, an increase in the EPS index corresponds to a reduction in per capita emissions. The effectiveness of these policies in decreasing CO_2_ emissions per capita is greater in countries with lower emissions (i.e., lower quantiles) compared to those with higher emissions, and it is more pronounced for PBA emissions than for CBA emissions. In terms of the Environmental Kuznets Curve (EKC) hypothesis, the results reported in Tables 8 and 9 reveal that the coefficients for economic growth – measured by GDP per capita (lnGDP) and its square (lnGDP2) – are both statistically significant and correctly signed. This indicates an inverted U-shaped relationship, confirming the EKC hypothesis in the selected countries across all quantiles and both CO_2_ emission measures. Moreover, the coefficients of emissions measured by CBA indicate a more pronounced positive relationship with GDP per capita, aligning with findings from previous studies (Karakaya et al. 2019; Fan et al. 2016; Papież et al. 2022b), particularly in the context of decoupling economic growth from CO_2_ emissions. Furthermore, the quantile approach employed in our study reveals that the relationship between income and emissions strengthens as the level of emissions increases. This suggests that the impact of economic growth on CO_2_ emissions is more significant in countries with higher per capita emissions.

Analyses of the relationships between a country’s vulnerability to climate change (lnGain) and CO_2_ emissions yield intriguing results. Our study reveals relatively ambiguous correlations between CO_2_ emissions and climate vulnerability Positive and statistically significant values are observed for smaller emitters (0.379 for the 10th quantile of PBA) and positive values for the 30th quantile of PBA (0.146). In contrast, larger emitters show negative and statistically significant coefficients for both measures of CO_2_ emissions, with values of −0.283 and −0.438 for the 70th and 90th quantiles of PBA, respectively, and −0.319, −0.422, and −0.513 for the 50th, 70th, and 90th quantiles of CBA, respectively. These findings indicate a decreasing trend in the coefficients as the quantiles increase, suggesting that the impact of the ND-GAIN index on emission reduction becomes more pronounced at higher emission levels. In other words, as per capita CO_2_ emissions rise, a country’s vulnerability to climate change is associated with lower emissions. This effect is particularly stronger when measuring CO_2_ emissions using CBA rather than PBA. Notably, in countries with lower emissions measured by PBA, an increase in climate vulnerability and adaptive capacity correlates with higher emissions.

The results for the globalization index (lnKOF) indicate its positive impact on environmental quality. All regression coefficients are negative and statistically significant for both measures of CO_2_ emissions. The impact of globalization on CO_2_ emissions is particularly pronounced for CBA emissions, with coefficients ranging from −0.485 to −0.540. The coefficients for PBA emissions range from −0.275 to −0.297. The direction of parameter change remains consistent across quantiles. The coefficients decrease as the quantiles increase, indicating that globalization’s impact on reducing emissions is more pronounced at higher emission levels. In other words, as CO_2_ emissions per capita rise, the expansion of globalization contributes to greater reductions in emissions. These findings align with those reported by Lv and Xu (2018), Rafindadi and Usman (2019), Tahir et al. (2021), and Aluko et al. (2021), who argue for the positive effects of globalization on environmental quality. Dinda (2004) posits that globalization might induce an environmental ‘race to the bottom’, which could reduce pollution by intensifying competition for investment and jobs. Consequently, poorer economies may enhance their environmental quality by investing in initiatives that boost income and employment. However, this view is challenged by other studies (Awan et al. 2020; Chishti et al. 2020; Le and Ozturk 2020; Leal and Marques 2020; Baloch et al. 2021; Wen et al. 2021). Bektaş and Ursavaş (2023) note that the expansion of global trade has led to increased CO_2_ emissions, particularly in developing countries. Our study contributes to this discourse by demonstrating that globalization positively affects environmental quality across both emission measures, particularly for the largest emitters and CBA emissions.

Tables 16, 17, 18, and 19 in the Appendix present the regression coefficients for four models incorporating three components of the EPS index, along with a control variable for both emission measures (PBA_Model 1_GAIN, PBA_Model 1_KOF, CBA_Model 1_GAIN, CBA_Model 1_KOF). First, these coefficients reaffirm the findings presented in Tables 6 and 7, indicating that the effectiveness of the three environmental policy instruments varies significantly. Market-based instruments demonstrate a substantial impact on CO_2_ emission reductions, while technology support policies have the least effect. Furthermore, the effectiveness of market-based and non-market-based instruments is more pronounced in countries with lower per capita CO_2_ emissions. Conversely, technology support policies exhibit a stronger impact in countries with higher per capita CO_2_ emissions. Second, the incorporation of the three components of the EPS index into the models does not fundamentally alter the effects of economic growth (lnGDP) and its square, nor the indices representing a country’s vulnerability to climate change (lnGain) and globalization (lnKOF) on the environment. These impacts remain very similar to those reported in Tables 8 and 9.

## Conclusions and policy implications

The study examines the impact of environmental regulations on CO_2_ emissions across countries with varying emission levels. Specifically, it analyzes three components of the Environmental Policy Stringency (EPS) index: market-based instruments, non-market-based instruments, and technology support. To assess the effectiveness of these policies in high, medium, and low-emission countries, the study employs the Moments-Quantile Regression (MMQR) method developed by Machado and Silva (2019). The key findings can be summarized as follows.

First, increased stringency in environmental regulations correlates with a reduction in CO_2_ emissions per capita. Notably, these regulations are more effective in countries with lower per capita emissions (i.e., lower quantiles) compared to those with higher per capita emissions. This trend is likely due to the stronger correlation between economic activity and CO_2_ emissions in high-emission countries, where a reduction in emissions may necessitate a decrease in economic activity. When comparing the impact of the EPS index on production-based (PBA) and consumption-based (CBA) emissions, it is evident that the effect is more pronounced in case of PBA emissions, which are typically used in official accounting systems and environmental legislation and regulations. While the effect on CBA emissions is smaller, it remains significant. This result is understandable, as current climate policies primarily target domestic actors’ adaptation to new regulations. However, this dynamic could change if climate policies incorporate instruments that influence external agents, such as environmental tariffs.

Second, all three types of environmental policies influence emissions, albeit in distinct ways. Among these regulations, market-based instruments prove to be the most effective in reducing CO_2_ emissions, while technology support instruments have the least impact. Notably, the effectiveness of both market-based and non-market-based instruments is greater in countries with relatively low per capita emissions. Conversely, policies that support research and development (R&D) and the deployment of wind and solar energy technologies tend to have a stronger impact in countries with higher pollution levels.

Third, increased globalization serves as a catalyst for countries to reduce their CO_2_ emissions. This effect is particularly pronounced in countries with higher per capita emissions and is more significant when considering consumption-based (CBA) rather than production-based (PBA) measures of CO_2_ emissions. In contrast, in countries with lower per capita emissions, a country’s vulnerability to climate change and its capacity to adapt to its adverse effects can lead to an increase in PBA emissions per capita. However, in countries with higher per capita CO_2_ emissions, this vulnerability and adaptive capacity significantly contribute to a reduction in both PBA and CBA emissions.

Our study has significant policy implications.

The stringency of environmental regulations has proven to be an effective tool for mitigating environmental pressures. This means that the environmental policies implemented so far – including certificates, taxes, and limits on CO_2_ emissions – substantially contribute to enhancing environmental quality by reducing pollution.

Among the three types of instruments examined, market-based policies that put a price on pollution have the most pronounced impact. Therefore, countries should prioritize and support ongoing efforts to implement CO_2_ trading schemes, renewable energy trading initiatives, and various forms of taxation. Our findings advocate for the broader adoption of legislative measures such as taxes, permits, and certificates, as these instruments are beneficial to the environment.

Non-market instruments, such as emission standards and limits, also play a crucial role in reducing emissions. Countries should ensure the establishment of stringent limits on permissible emissions of sulfur oxides, nitrogen oxides, and CO_2_ from new large coal-fired power plants, as well as impose restrictions on sulfur content in diesel fuel for cars.

These measures will positively impact CO_2_ emissions reduction in all countries, particularly those with lower per capita emissions. However, for the largest emitters, it is advisable to complement these policies with technological support, which can yield additional environmental benefits. Thus, countries with the highest CO_2_ emissions should focus on developing innovative clean technologies and investing in renewable energy sources, particularly wind and solar power.

Moreover, given the strong correlation between living standards (as measured by GDPpc) and emissions in countries with higher CO_2_ output, policymakers in these countries should prioritize strategies for emission reduction. It is crucial for these policymakers to tailor their approaches according to specific emission levels. In heavily polluting countries, policies should be designed with their high emitter status in mind, focusing on transitioning toward a low-carbon economy while ensuring economic stability. This can be accomplished by strengthening market-based instruments, which have the most significant impact on CO_2_ reduction. For example, increasing the stringency of carbon pricing mechanisms or introducing new ones can effectively incentivize emission reductions.

## Data Availability

Data will be made available on request.

## Appendix

See Table 10.

**Table 10:** Descriptive statistics in 1995 and 2020

Year	1995				2020			
Measure	min	mean	max	standard deviation	min	mean	max	standard deviation
PBApc	0.79	9.07	22.40	4.64	1.75	6.95	15.59	3.26
CBApc	0.75	10.10	22.40	4.75	1.63	8.09	15.68	3.34
GDPpc	618.10	24,096.20	74,896.10	17,801.93	1796.00	34,676.00	104,128.00	23,581.59
EPS	0.00	0.87	2.33	0.61	0.83	2.97	4.89	0.99
Nm_EPS	0.00	1.09	3.25	0.94	1.50	4.96	6.00	1.24
M_EPS	0.00	0.75	2.50	0.51	0.17	1.74	4.17	1.01
Ts_EPS	0.00	0.77	3.00	0.80	0.00	2.22	6.00	1.47
KOF	39.49	67.75	84.53	11.98	62.76	80.91	90.61	7.57
Gain	40.05	57.96	68.70	7.07	43.98	63.07	75.41	7.31

See Table 11

**Table 11:** Pesaran’s cross-sectional dependence (CD) test (Pesaran 2021, 2015)

Variables	CD-test	Corr- average correlation coefficient	Abs Corr—average absolute correlation coefficient
lnPBApc	43.413***	0.32	0.69
lnCBApc	36.389***	0.27	0.54
lnGDPpc	115.098***	0.85	0.85
lnEPS	112.279***	0.84	0.84
lnNm_EPS	113.697***	0.84	0.84
lnM_EPS	48.313***	0.36	0.51
lnTs_EPS	64.430***	0.48	0.50
lnGain	107.852***	0.80	0.80
lnKOF	128.105***	0.95	0.95

***, **, * denote a significance level of 1, 5 and 10% respectively

See Table 12

**Table 12:** Pesaran and Yamagata (2008) test for homogeneity of slopes in the baseline models

Test statistics/ Model	PBA_Model 0	PBA_Model 1	CBA_Model 0	CBA_Model 1
Delta tilde	29.677***	23.719***	26.547***	22.859***
Delta tilde adjusted	33.022***	27.747***	29.538***	26.740***

***, **, * denote a significance level of 1, 5 and 10% respectively

See Table 13

**Table 13:** Pesaran and Yamagata (2008) test for homogeneity of slopes in the models with control variables

Test statistics/ Model	PBA_Model 0_GAIN_KOF	PBA_Model 0_GAIN_KOF	PBA_Model 1_KOF	PBA_Model 1_GAIN	CBA_Model 1_KOF	CBA_Model 1_GAIN
Delta tilde	28.296***	22.258***	22.443***	22.258***	20.676***	21.370***
Delta tilde adjusted	32.262***	26.750***	26.973***	26.750***	24.850***	25.684***

***, **, * denote a significance level of 1, 5 and 10% respectively

See Table 14

**Table 14:** Pesaran’s (2007) panel unit root CIPS test

Variables	Level Zt-bar	First difference Zt-bar	Level Zt-bar	First difference Zt-bar
	**With an intercept and without trend**		**With an intercept and a linear trend**	
lnPBApc	4.365	-11.227***	-0.595	-7.626***
lnCBApc	4.623	-8.628***	1.057	-5.715***
lnGDPpc	-2.468***/ 0.677(0)	-4.393***	0.248	-1.224/-4.513(0)***
lnEPS	-5.6***	-12.094***	-2.956***/0.176(3)	-9.002***
lnNm_EPS	-4.112***/-0.801(5)	-12.576***	-4.116***/-0.820(3)	-9.362***
lnM_EPS	-0.229	-11.731***	1.868	-9.568***
lnTs_EPS	-3.241***/-0.833(3)	-10.551***	-0.101	-8.197***
lnGain	-3.605***/-0.876(5)	-11.371***	-0.932	-8.77***
lnKOF	-2.084***/ 0.227(3)	-9.707***	-1.532*/0.238(2)	-7.799***

*, **, *** indicate the level of significance at 10, 5, and 1%, respectively; Pesaran’s (2007) test is performed using the Stata ‘ multipurt ‘ command

See Table 15

**Table 15:** Pedroni’s (2004, 1999) and Westerlund’s (2005) cointegration tests

	lnPBA		lnCBA	
	Model 0	Model 1	Model 0	Model 1
Pedroni (1999, 2004)				
Modified Phillips-Perron t	1.7823**	3.9683***	0.9266	3.8750***
Phillips-Perron t	-2.4042***	-2.4577***	-4.1893***	-3.3828***
Augmented Dickey-Fuller t	-3.0767***	-3.0289***	-3.5109***	-2.6098***
Westerlund (2005)				
Variance ratio (some panels)	-2.507***	-1.448*	-2.775***	-1.763**
Variance ratio (all panels)	-1.733**	-1.096	-1.729**	-0.496
Westerlund (2007) test				
Gt	-3.306**	-3.523**	-3.19**	-3.404**
Ga	-10.396*	-6.725*	-9.774	-5.733
Pt	-13.158	-12.915	-17.491*	-17.99**
Pa	-9.157	-7.481	-11.556**	-9.75**

***, **, * denote a significance level of 1, 5 and 10% respectively;

See Table 16

**Table 16:** PBA_Model 1_KOF (Quantile Regression)

VARIABLES	location	scale	qtile_10	qtile_30	qtile_50	qtile_70	qtile_90
lnGDPpc	3.562***	-0.344***	4.098***	3.796***	3.495***	3.280***	3.093***
	(19.83)	(-3.299)	(16.72)	(19.65)	(19.06)	(16.48)	(13.58)
lnGDPpc2	-0.167***	0.021***	-0.199***	-0.181***	-0.163***	-0.150***	-0.138***
	(-15.83)	(3.457)	(-14.35)	(-16.33)	(-15.02)	(-12.59)	(-10.14)
lnNm_EPS	-0.0514***	0.0133	-0.072***	-0.060***	-0.049***	-0.041**	-0.033*
	(-2.910)	(1.357)	(-2.586)	(-2.836)	(-2.855)	(-2.492)	(-1.901)
lnM_EPS	-0.190***	-0.002	-0.187***	-0.189***	-0.191***	-0.192***	-0.193***
	(-6.438)	(-0.118)	(-4.451)	(-5.669)	(-6.539)	(-6.459)	(-5.884)
lnTs_EPS	-0.047***	-0.018**	-0.019	-0.035*	-0.051***	-0.062***	-0.072***
	(-3.033)	(-2.054)	(-0.811)	(-1.947)	(-3.291)	(-3.987)	(-4.129)
lnKOF	-0.391***	-0.0717	-0.279*	-0.342***	-0.405***	-0.449***	-0.488***
	(-3.836)	(-1.263)	(-1.790)	(-2.841)	(-4.062)	(-4.599)	(-4.533)
Constant	-14.82***	1.739***	-17.53***	-16.00***	-14.49***	-13.40***	-12.45***
	(-21.34)	(4.140)	(-16.70)	(-20.07)	(-20.57)	(-18.60)	(-15.26)

z-statistics in parentheses *** *p* < 0.01, ** *p* < 0.05, * *p* < 0.1

See Table 17

**Table 17:** PBA_Model 1_GAIN (Quantile Regression)

VARIABLES	location	scale	qtile_10	qtile_30	qtile_50	qtile_70	qtile_90
lnGDPpc	3.333***	-0.406***	3.951***	3.619***	3.263***	2.992***	2.786***
	(18.24)	(-4.329)	(16.47)	(18.38)	(17.53)	(15.26)	(12.79)
lnGDPpc2	-0.157***	0.024***	-0.194***	-0.174***	-0.153***	-0.137***	-0.124***
	(-13.99)	(4.388)	(-13.88)	(-14.81)	(-13.25)	(-11.09)	(-9.050)
lnNm_EPS	-0.071***	0.020**	-0.101***	-0.085***	-0.068***	-0.055***	-0.045***
	(-4.144)	(2.196)	(-3.903)	(-4.193)	(-4.065)	(-3.396)	(-2.594)
lnM_EPS	-0.185***	0.0119	-0.203***	-0.193***	-0.182***	-0.174***	-0.168***
	(-6.271)	(0.797)	(-4.679)	(-5.623)	(-6.341)	(-6.241)	(-5.641)
lnTs_EPS	-0.053***	-0.021**	-0.021	-0.038**	-0.056***	-0.071***	-0.081***
	(-3.480)	(-2.334)	(-0.850)	(-2.043)	(-3.822)	(-5.023)	(-5.255)
lnGain	-0.156	-0.243***	0.215	0.0157	-0.198	-0.360***	-0.483***
	(-1.324)	(-3.734)	(1.388)	(0.126)	(-1.637)	(-2.754)	(-3.211)
Constant	-14.53***	2.667***	-18.60***	-16.42***	-14.07***	-12.29***	-10.94***
	(-15.60)	(5.343)	(-14.19)	(-15.77)	(-14.79)	(-12.70)	(-10.19)

z-statistics in parentheses *** *p* < 0.01, ** *p* < 0.05, * *p* < 0.1

See Table 18

**Table 18:** CBA_Model 1_KOF (Quantile Regression)

VARIABLES	location	scale	qtile_10	qtile_30	qtile_50	qtile_70	qtile_90
lnGDPpc	3.304***	-0.361***	3.858***	3.554***	3.246***	3.017***	2.794***
	(17.37)	(-3.136)	(14.75)	(17.08)	(16.75)	(14.26)	(11.13)
lnGDPpc2	-0.149***	0.0212***	-0.182***	-0.164***	-0.146***	-0.132***	-0.119***
	(-12.87)	(3.121)	(-11.55)	(-12.97)	(-12.36)	(-10.34)	(-7.894)
lnNm_EPS	-0.0228	0.0264***	-0.0633**	-0.0411*	-0.0185	-0.00171	0.0146
	(-1.274)	(2.637)	(-2.255)	(-1.904)	(-1.058)	(-0.103)	(0.800)
lnM_EPS	-0.134***	-0.00833	-0.122***	-0.129***	-0.136***	-0.141***	-0.146***
	(-4.399)	(-0.543)	(-3.164)	(-3.998)	(-4.423)	(-4.258)	(-3.843)
lnTs_EPS	-0.0179	-0.0183*	0.0102	-0.00522	-0.0208	-0.0324*	-0.0437**
	(-0.993)	(-1.945)	(0.391)	(-0.253)	(-1.168)	(-1.833)	(-2.227)
lnKOF	-0.642***	-0.0751	-0.526***	-0.590***	-0.654***	-0.701***	-0.748***
	(-6.169)	(-1.307)	(-4.244)	(-5.658)	(-6.161)	(-5.776)	(-5.161)
Constant	-12.89***	1.882***	-15.78***	-14.19***	-12.59***	-11.39***	-10.23***
	(-17.54)	(4.057)	(-14.68)	(-16.92)	(-16.77)	(-14.26)	(-10.73)

z-statistics in parentheses *** *p* < 0.01, ** *p* < 0.05, * *p* < 0.1

See Table 19

**Table 19:** CBA_Model 1_GAIN (Quantile Regression)

VARIABLES	location	scale	qtile_10	qtile_30	qtile_50	qtile_70	qtile_90
lnGDPpc	2.896***	-0.405***	3.533***	3.179***	2.850***	2.579***	2.320***
	(13.90)	(-3.710)	(13.16)	(14.21)	(13.46)	(11.45)	(8.946)
lnGDPpc2	-0.130***	0.0229***	-0.166***	-0.146***	-0.128***	-0.112***	-0.0978***
	(-10.03)	(3.381)	(-10.22)	(-10.67)	(-9.660)	(-7.875)	(-5.902)
lnNm_EPS	-0.0507***	0.0326***	-0.102***	-0.0735***	-0.0470***	-0.0252	-0.00442
	(-2.867)	(3.409)	(-3.964)	(-3.598)	(-2.654)	(-1.441)	(-0.224)
lnM_EPS	-0.123***	0.00700	-0.134***	-0.128***	-0.122***	-0.118***	-0.113***
	(-4.078)	(0.440)	(-3.297)	(-3.876)	(-4.061)	(-3.698)	(-3.107)
lnTs_EPS	-0.0238	-0.0259***	0.0169	-0.00572	-0.0268	-0.0441**	-0.0607***
	(-1.336)	(-2.797)	(0.651)	(-0.276)	(-1.507)	(-2.553)	(-3.201)
lnGain	-0.478***	-0.118	-0.292	-0.396**	-0.492***	-0.571***	-0.646***
	(-2.905)	(-1.346)	(-1.281)	(-2.143)	(-3.002)	(-3.367)	(-3.323)
Constant	-11.50***	2.301***	-15.12***	-13.11***	-11.24***	-9.702***	-8.230***
	(-9.951)	(3.975)	(-9.658)	(-10.13)	(-9.671)	(-8.258)	(-6.217)

Note: z-statistics in parentheses *** *p* < 0.01, ** *p* < 0.05, * *p* < 0.1

See Fig. 5

## Footnotes

1 Due to the limitations of the Westerlund cointegration test regarding the number of variables, we cannot consider a model with both control variables.

2 Other test results for models with control variables are available on request.
